# Internet-Delivered Acceptance and Commitment Therapy for Anxiety Treatment: Systematic Review

**DOI:** 10.2196/12530

**Published:** 2019-01-29

**Authors:** Joshua Kelson, Audrey Rollin, Brad Ridout, Andrew Campbell

**Affiliations:** 1 Cyberpsychology Research Group Faculty of Health Sciences The University of Sydney Sydney Australia

**Keywords:** anxiety, anxiety disorders, acceptance and commitment therapy, mindfulness, telemedicine, internet, e-therapy

## Abstract

**Background:**

Anxiety conditions are debilitating and prevalent throughout the world. Acceptance and Commitment Therapy (ACT) is an effective, acceptance-based behavioral therapy for anxiety. However, there are treatment barriers (eg, financial, geographical, and attitudinal), which prevent people from accessing it. To overcome these barriers, internet-delivered ACT (iACT) interventions have been developed in recent years. These interventions use websites to deliver ACT information and skill training exercises on the Web, either as pure self-help or with therapist guidance.

**Objective:**

This systematic review aimed to examine the therapeutic impact of iACT on all anxiety conditions.

**Methods:**

The EMBASE, MEDLINE, ProQuest Central, PsycINFO, Scopus, and Web of Science databases were searched up to September 2018. The titles and abstracts of remaining records after deduplication were screened by 2 authors with a total of 36 full-text articles being retained for closer inspection next to eligibility criteria. Empirical studies of all designs, population types, and comparator groups were included if they appraised the impact of iACT treatment on any standardized measure of anxiety. Included studies were appraised on methodological quality and had their data extracted into a standardized coding sheet. Findings were then tabulated, and a narrative synthesis was performed because of the heterogeneity found between studies.

**Results:**

A total of 20 studies met inclusion criteria. There were 11 randomized controlled trials (RCTs) and 9 uncontrolled pilot studies. Participants across all studies were adults. The anxiety conditions treated were as follows: generalized anxiety disorder (GAD), social anxiety disorder (SAD), illness anxiety disorder (IAD), and general anxiety symptoms, with or without comorbid physical and mental health problems. A total of 18 studies reported significant anxiety reduction after iACT treatment. This was observed in studies that delivered iACT with (n=13) or without (n=5) therapist guidance. The average attrition rate across all included studies during the active iACT treatment phase was 19.19%. In the 13 studies that assessed treatment satisfaction, participants on average rated their iACT experience with above average to high treatment satisfaction.

**Conclusions:**

These findings indicate that iACT can be an efficacious and acceptable treatment for adults with GAD and general anxiety symptoms. More RCT studies are needed to corroborate these early iACT findings using empirical treatments in active control groups (eg, internet-delivered cognitive behavioral therapy). This would potentially validate the promising results found for SAD and IAD as well as address the full spectrum of anxiety disorders.

## Introduction

### Background

Anxiety and its related conditions are highly prevalent on a global scale [1]. The *Diagnostic and Statistical Manual of Mental Disorders-Fifth Edition* (DSM-5) describes multiple anxiety disorders such as specific phobia, generalized anxiety disorder (GAD), social anxiety disorder (SAD), illness anxiety disorder (IAD), panic disorder, agoraphobia, separation anxiety disorder, substance or medication-induced anxiety disorder, and selective mutism [2]. These disorders can start early in life [3], persist long after onset [4], and be highly debilitating from even subclinical symptomatology [5,6]. They are often comorbid with other mental health issues [7], and risk factors for suicide [8].

A potential solution for people with anxiety is Acceptance and Commitment Therapy (ACT)—a psychological treatment that teaches mindfulness skills to help people accept their anxiety and commit to living in accordance with personal values [9]. The primary aim of ACT is not to eliminate anxiety symptoms but rather to improve psychological flexibility which refers to a person’s ability to contact the present moment more fully as a conscious human being, and engage in values-based action [9,10]. This is accomplished in therapy by targeting 6 core processes of change: acceptance, contact with the present moment, cognitive defusion, self-as-context, values, and committed action [9]. ACT delivery is not constrained to one specific method or technology, but can make use of a host of methods and modalities to facilitate these processes [10]. Even though anxiety symptom reduction is not the primary focus of ACT, substantial evidence has accrued showing that it can significantly reduce anxiety symptoms and put anxiety disorders into remission [11-15]. In contrast to other established treatments for anxiety, such as cognitive behavioral therapy (CBT) [16,17] and pharmacotherapy [18], the ACT model provides a unique transdiagnostic approach [19] that emphasizes acceptance, mindfulness, and values-guided behavioral exercises, rather than control, logical analysis, and cognitive disputation exercises [10].

To make psychological treatment accessible to the broader population, researchers have developed Web-based therapy interventions [20]. This therapy involves using websites to provide mental health information and skill training online [21]. These interventions can overcome many of the structural and attitudinal barriers of face-to-face treatment such as therapist worker shortages, geographical distance, wait-lists, social stigma, financial cost, and work commitments [22], which are especially pronounced in rural areas [23]. Evidence indicates that Web-based interventions can be an effective, an acceptable, and a practical health care option for anxiety sufferers when used as pure self-help or as an adjunct to treatment-as-usual [24,25]. However, efforts to treat anxiety through the Web have largely been concentrated on CBT [26-29]. This is because the CBT tradition has a larger evidence base from a longer research history than the more recent ACT model [15,30]. Nevertheless, attrition and treatment adherence remain key issues for internet-delivered CBT (iCBT) [31,32]. It is therefore important to assess the potential benefits of other therapy models delivered through the Web, such as ACT. A previous review of randomized controlled trial (RCT) evidence indicated that internet-delivered ACT (iACT) can reduce anxiety and depression symptoms [33]. However, none of the RCTs published in this earlier review specifically targeted anxiety disorders nor examined the use of iACT for anxiety in other research designs.

### Objective

The aim of this systematic review was to provide a comprehensive and up-to-date account on the empirical status of iACT for anxiety. To this end, all studies that investigated the impact of iACT on any standardized measure of anxiety were appraised. This included pilot studies as they can cost-effectively determine the feasibility of new interventions and research pursuits before expending greater resources on developing complex interventions, or testing advanced research designs [34]. As iACT for anxiety is a nascent research field [33], including the following information can help: (1) inform researchers on what has already been done, and where treatment gaps for anxiety are present; (2) clarify what interventions hold the potential for acceptable, efficacious, and practical anxiety treatment; and (3) potentially guide the design, development, and empirical validation of new iACT interventions as either self-help or as an adjunct to treatment-as-usual.

## Methods

### Eligibility Criteria

This systematic review was performed using the Preferred Reporting Items for Systematic Reviews and Meta-Analyses (PRISMA) guidelines. A PRISMA checklist is available in Multimedia Appendix 1. To be included in the review, articles needed to report on empirical data obtained from iACT treatment on any standardized measure of anxiety. This criterion was set to ensure the validity and reliability of the anxiety symptom scores obtained at pre- and post-iACT treatment. Furthermore, to ensure that the review was comprehensive, all empirical research designs were included, as well as all population and comparator types. The iACT intervention under investigation needed to be delivered through a website and be based on at least 2 of the 6 core ACT processes: acceptance, contact with the present moment, cognitive defusion, self-as-context, values, and committed action. This criterion was set in accordance with Swain et al’s [14] reasoning that setting a minimum of 2 ACT processes will include interventions that address multiple aspects of the ACT model (rather than just a stand-alone technique), while simultaneously avoiding the reiteration of earlier review research on single processes (eg, pure mindfulness). Furthermore, studies emphasizing stress reduction (rather than anxiety reduction) or cognitive restructuring techniques were excluded in the present review [14]. Only articles written in the English language and published in peer-reviewed journals were included.

### Search Strategy

The following electronic databases of scientific research were searched: EMBASE, MEDLINE, ProQuest Central, PsycINFO, Scopus, and Web of Science. Keywords used to search each database were ((Anxiety) AND ((ACT) OR (acceptance and commitment therapy) OR (iACT)) AND ((Internet) OR (online) OR (web-based) OR (etherapy))). The search was limited to peer-reviewed papers published in English up to the month of September 2018. The reference lists of included papers were manually searched. The Association for Contextual Behavioral Science (ACBS) website [35] was also surveyed for iACT research. The ACBS organization is a worldwide online learning and research community for ACT.

### Article Selection

Upon removal of duplicate citations, article titles and abstracts were then independently screened and appraised next to the eligibility criteria by 2 researchers (JK and AR). Full text review was again conducted by both reviewers, and divergent views were resolved through discussion and mutual agreement.

### Data Extraction

Data from included studies were extracted by one reviewer (JK) into a standardized coding sheet and then checked by a second reviewer (AC). Data items extracted for synthesis included the following:

1. Reference source: first author surname and year of publication.
2. Anxiety-related problem/disorder under investigation.
3. Study design: methodology, comparator trial arms, and measurement points.
4. Population: country, participants, sample size, age, and gender breakdown.
5. iACT intervention details: intervention name, manual protocol, number of modules, treatment length, therapist guidance, and educational content.
6. Efficacy: study quality rating, anxiety measures, outcomes, and effect sizes.
7. Adherence: attrition rate, treatment satisfaction, and intention-to-treat analysis.

Attrition in this review was defined and measured as the relative number of participants who commenced but did not complete the active iACT treatment phase.

### Quality Assessment

Öst’s [36] *psychotherapy outcome study methodology rating form* (POMRF) was used to appraise the quality of the reviewed iACT articles. It is a 22-item questionnaire that addresses key methodological issues pertinent to psychotherapy intervention studies such as research design, participant details, psychotherapy implementation, therapist characteristics, user adherence, statistical analyses, and clinical significance [36]. It can be used to assess study quality across multiple research designs, including RCTs, and has been used in an earlier systematic review by Swain et al [14] on ACT for anxiety. Each item in the POMRF is rated on a 3-point scale ranging from 0 (*poor*) to 2 (*good*), with the total POMRF score calculated as the sum of all points. The total score for any given study can range from 0 to 44 points, with higher scores indicating greater overall methodological quality. Öst [36] found the POMRF questionnaire to demonstrate high internal consistency (Cronbach alpha of 0.86) and interrater reliability (kappa coefficient mean of 0.75). In this systematic review, the included iACT studies were independently assessed next to the POMRF items by 2 reviewers (JK and AC) who individually calculated the overall POMRF scores. On completion, these scores were compared between reviewers, and the discrepancies were resolved by discussion and mutual agreement.

### Narrative Synthesis

A narrative synthesis approach was chosen for this systematic review due to a paucity of iACT papers and the heterogeneity found between studies (eg, populations, designs, comparators, and anxiety outcomes). Results are presented as descriptive data with no further analyses performed.

## Results

### Study Selection

**Figure 1:**
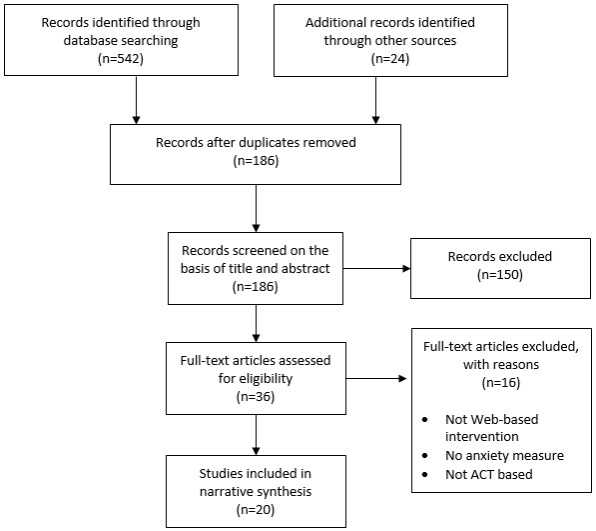
Flowchart of systematic review search results. ACT: Acceptance and Commitment Therapy.

The literature search yielded 566 papers. A total of 186 records remained after duplicate removal. Of these records, 20 met the eligibility criteria (see Figure 1).

### Participant Characteristics

Table 1 shows that the majority of studies and their participants were from Sweden (35%, 7/20) and the United States (35%, 7/20), with the remaining studies from Australia (10%, 2/20), the Netherlands (10%, 2/20), Finland (5%, 1/20), and Denmark (5%, 1/20). All participants were adults with mean ages ranging from 18.37 to 59 years. Sample sizes ranged from 13 to 238 participants, with a median of 76 participants. Participants were mainly female with an average sample proportion of 72.62% (range 43.4%-100%). Both clinical (65%, 13/20) and nonclinical (35%, 7/20) studies were conducted. Anxiety symptom reduction was typically measured as a secondary outcome with 15 out of 20 studies (75%) targeting people with a general health issue and/or comorbid anxiety problem. Several studies (25%, 5/20) targeted a specific anxiety disorder. Diagnoses of GAD and SAD were obtained by clinical interviews delivered in person or by phone, whereas IAD diagnoses were obtained by videoconferencing.

### Research Designs

Table 2 shows that just over half of the studies were RCTs (55%, 11/20). Control groups consisted of wait-lists (n=8), online discussion forums (n=2), expressive writing (n=2), iCBT program (n=1), and a mental health education website (n=1). The remaining studies in the review were uncontrolled pilot studies (45%, 9/20). There were 12 studies that reported between-group analyses, and 14 that reported within-group analyses, on their respective anxiety measures. Pretest, posttest, and follow-up assessments were used in 14 out of 20 studies (70%). The follow-up assessments ranged from 2 weeks to 1 year, with a median of 3 months.

### Internet-Delivered Acceptance and Commitment Therapy Interventions

Educational content was typically presented in multimedia formats (ie, audio, video, text, images, and animations) with interactive exercises, worksheets, quizzes, and homework assignments provided (see Table 3). Several studies (40%, 8/20) based their content directly upon a detailed treatment manual that is publicly available. Every study appraised the iACT treatment of individuals, as no group-based approaches were adopted. Treatment lengths ranged from 2 to 12 weeks, with a mode of 8. The number of treatment modules ranged from 2 to 9, with a mode of 7. Most studies (70%, 14/20) tested iACT with therapist guidance, except for several (30%, 6/20) which delivered iACT as pure self-help.

### Attrition and Treatment Satisfaction

Attrition in the active iACT treatment phase varied between the studies, with a mean of 19.19% and a range of 0 to 60.77% (see Table 4). To handle missing data, 18 out of 20 studies reported the use of an intention-to-treat analysis (90%). Several studies (55%, 9/20) used a standardized measure to collect treatment satisfaction ratings from participants after iACT completion. These measures included the Client Satisfaction Questionnaire-8 items (CSQ-8) and System Usability Scale (SUS). Several studies (35%, 7/20) incorporated nonstandardized questions (NSQ) on treatment satisfaction. Participants’ mean rating of their iACT experience ranged from above average to high satisfaction.

### Anxiety Measures

Details of the standardized anxiety measures used are summarized in Table 5. The anxiety subscales of the Depression Anxiety Stress Scales-21 items and Hospital Anxiety and Depression Scale were the most frequently used measures (7 and 5 studies, respectively). Most studies (70%, 14/20) used a single anxiety measure, whereas the remaining 6 studies (30%) used multiple measures.

### Anxiety Outcomes

Nearly all studies (90%, 18/20) reported small-to-large improvement on anxiety symptoms after treatment (see Table 5). Two studies on GAD found large anxiety reductions immediately after iACT, and at the 3- [40] and 6-month [39] follow-up points. Two studies on SAD found large reductions in anxiety symptoms after 8 weeks of iACT and at 3-month follow-up [42,43]. Hoffmann et al [45] found large effect sizes on both anxiety measures at posttest and 3-month follow-up for IAD. Anxiety symptoms also reduced for people with fibromyalgia [51], tinnitus [44], chronic pain [37,55], major depression [38], a history of interpersonal trauma [41], and mild to moderate symptoms of depression, anxiety, and stress [46-48,50,53,54,56].

However, the study by Levin et al [49] found no significant subclinical anxiety reduction after 3 weeks of self-help iACT treatment. Similarly, Räsänen et al [54] detected no significant difference between an iACT and a wait-list control group on anxiety symptoms among university students. Furthermore, Molander et al [52] found no significant difference in anxiety between their iACT and wait-list control group at post-treatment among adults with hearing problems. When comparing iACT to iCBT on anxiety reduction in tinnitus sufferers, Hesser et al [44] found no significant differences. No significant differences were found between iACT with or without therapist support for people with SAD [43]. Finally, the study by Trompetter et al [55] found no significant difference between iACT and expressive writing for reducing anxiety symptoms in chronic pain sufferers.

### Quality Assessment

Total POMRF scores for each study are presented in Table 5. Out of a possible total of 44 points, the mean score for the reviewed iACT studies was 19 (SD 5.7), with a range of 10 to 29. The included studies gave either fair (65%, 13/20) or good (35%, 7/20) descriptions on their research participants in terms of inclusion/exclusion criteria, demographics, and comorbidity.

**Table 1:** Description of participants in reviewed internet-delivered Acceptance and Commitment Therapy (iACT) studies.

Source	Country	Problem/diagnosis	Clinical	N^a^	Age (years), mean (SD)	Females in total sample, n (%)
Buhrman [37]	Sweden	Chronic pain	Yes	76	49.1 (10.34)	45 (59)
Carlbring [38]	Sweden	Major depression	Yes	80	44.4 (13.5)	66 (82)
Dahlin [39]	Sweden	GAD^b^	Yes	103	39.48 (10.73)	86 (83.5)
Dahlin [40]	Sweden	GAD	Yes	14	32 (10)	11 (78)
Fiorillo [41]	United States	Interpersonal trauma	Yes	25	39.12 (16)	25 (100)
Gershkovich [42]	United States	SAD^c^	Yes	13	33.2 (10.04)	9 (69)
Gershkovich [43]	United States	SAD	Yes	42	31.5 (9.95)	27 (64)
Hesser [44]	Sweden	Tinnitus	Yes	99	48.5 (14.7)	43 (43)
Hoffmann [45]	Denmark	IAD^d^	Yes	15	38.8 (NR^e^)	12 (67)
Kelson [46]	Australia	DAS^f^ symptoms	No	40	21.62 (2.4)	21 (52)
Levin [47]	United States	DAS symptoms	No	76	18.37 (.54)	41 (54)
Levin [48]	United States	DAS symptoms	No	82	21.88 (3.50)	62 (75)
Levin [49]	United States	DAS symptoms	No	234	21.61 (5.48)	180 (77)
Levin [50]	United States	DAS symptoms	No	79	20.51 (2.73)	52 (66)
Ljótsson [51]	Sweden	Fibromyalgia	Yes	41	52 (9)	41 (100)
Molander [52]	Sweden	Hearing problems	Yes	61	59 (11)	41 (67.2)
Pots [53]	The Netherlands	Depressive symptoms	Yes	236	46.85 (12.06)	179 (75.8)
Räsänen [54]	Finland	DAS symptoms	No	68	24.29 (3.28)	58 (85.3)
Trompetter [55]	The Netherlands	Chronic pain	Yes	238	52.8 (12.37)	181 (76)
Viskovich [56]	Australia	DAS symptoms	No	130	26.3 (7.96)	98 (75)

^a^Refers to total number of participants.

^b^GAD: generalized anxiety disorder.

^c^SAD: social anxiety disorder.

^d^IAD: Illness anxiety disorder.

^e^NR: not reported.

^f^DAS: depression, anxiety, and stress.

**Table 2:** Description of research designs in reviewed internet-delivered Acceptance and Commitment Therapy (iACT) studies.

Source	RCT^a^	Trial arms (n)^b^	Design	Measurements
Buhrman [37]	Yes	iACT (38); forum (38)	BG^c^	Pre^d^, Post^e^, 6-month FU^f^
Carlbring [38]	Yes	iACT (40); WLC^g^ (40)	BG	Pre, Post, 3-month FU
Dahlin [39]	Yes	iACT (52); WLC (51)	WG^h^; BG	Pre, Post, 6-month FU
Dahlin [40]	No	iACT (14)	WG	Pre, Post, 2 to 3-month FU
Fiorillo [41]	No	iACT (25)	WG	Pre, Post
Gershkovich [42]	No	iACT (13)	WG	Pre, Mid^i^, Post, 3-month FU
Gershkovich [43]	No	iACT (22); iACT + TS^j^ (20)	WG, BG	Pre, Mid, Post
Hesser [44]	Yes	iACT (35); iCBT^k^ (32); forum (32)	BG	Pre, Post, 1-year FU
Hoffmann [45]	No	iACT (15)	WG	Pre, Post, 3-month FU
Kelson [46]	No	iACT (40)	WG	Pre, Post, 2-week FU
Levin [47]	Yes	iACT (37); WLC (39)	WG; BG	Pre, Post, 3-week FU
Levin [48]	No	iACT (82)	WG	Pre, Post
Levin [49]	Yes	iACT (114); website (120)	WG; BG	Pre, Post, 3-month FU
Levin [50]	Yes	iACT (40); WLC (39)	WG; BG	Pre, Post
Ljótsson [51]	No	iACT (41)	WG	Pre, Post, 6-month FU
Molander [52]	Yes	iACT (31); WLC (30)	BG	Pre, Post
Pots [53]	Yes	iACT (82); EW^l^ (67); WLC (87)	BG	Pre, Post, 3 and 6-month FU
Räsänen [54]	Yes	iACT (33); WLC (35)	WG; BG	Pre, Post, 1-year FU
Trompetter [55]	Yes	iACT (82); EW (79); WLC (77)	BG	Pre, Post, 3-month FU
Viskovich [56]	No	iACT (130)	WG	Pre, Post

^a^RCT: randomized controlled trial.

^b^Refers to number of participants.

^c^BG: between-groups.

^d^Pre: pretest.

^e^Post: posttest.

^f^FU: follow-up.

^g^WLC: wait-list control.

^h^WG: within-groups.

^i^Mid: midtest.

^j^TS: therapist support.

^k^iCBT: internet-delivered cognitive behavioral therapy.

^l^EW: expressive writing.

All studies’ participants were either somewhat or very representative of people seeking treatment for their respective anxiety-related issue. Diagnostic reliability was obtained in all 13 clinical studies with a structured interview from a trained assessor. Several studies (40%, 8/20) recruited participants with anxiety conditions inclusive of both acute chronicity (ie, less than one year duration) and low severity. Of the remaining studies, 5 consisted of chronic anxiety sufferers with at least moderate severity [37,41,45,51,55], and the other 7 included people with subthreshold symptoms [46-48,50,54,56].

Most studies (70%, 14/20) were rated poorly on research design because of being uncontrolled or only making use of a wait-list control group (see Table 2). The control for concomitant treatments outside of iACT was also generally poor (65%, 13/20). Several studies (45%, 9/20) reported a power analysis. Just over half of the studies (60%, 12/20) randomly assigned participants to treatment conditions, with 6 of these also randomly assigning therapists [38,39,44,53-55]. Assessment points were typically conducted at pretest, posttest, and follow-up of less than a year (60%, 12/20). Two studies had follow-up of a year or more [44,54]. All iACT studies employed adequate statistical analyses and presented results fully with means and SDs.

All studies incorporated moderate or very specific anxiety outcome measures with good psychometric properties for their targeted conditions. Details on the clinical significance of therapy outcomes were provided in several studies (45%, 9/20), 5 of which used Jacobson and Truax’s [57] criteria for clinically significant change [39,41,43,44,53]. There was no blinding of assessors across any included iACT studies. Outcome measures were collected via the internet in the form of self-report questionnaires.

About 8 of the iACT interventions under investigation were based on manual treatment programs (see Table 3). All therapist-guided iACT studies (70%, 14/20) deployed at least 2 therapists. Graduate psychology students were often used to guide participants through the iACT interventions (55%, 11/20). Therapist competence was checked either somewhat or frequently in all guided iACT studies. The equality of therapy hours between conditions was good in the 3 studies that had an active treatment control [44,53,55]. Treatment adherence checks were made in all studies and proportions of attrition rates were provided. Intent-to-treat analyses were performed in all but 2 studies (see Table 4).

**Table 3:** Details of the internet-delivered Acceptance and Commitment Therapy (iACT) interventions studied.

Source	Name	Manual	Modules	Length (weeks)	Guided	System features
Buhrman [37]	Not specified	No	7	7	Yes	Audio, text, interactive exercises
Carlbring [38]	The Depression Help	No	7	8	Yes	Multimedia^a^, animations, interactive exercises, homework, CD
Dahlin [39]	The Worry Help	No	7	9	Yes	Multimedia, animations, interactive exercises, homework, CD
Dahlin [40]	The Worry Help	No	7	8-10	Yes	Multimedia, animations, interactive exercises, homework, CD
Fiorillo [41]	Not specified	Yes	6	6	No	Multimedia, interactive exercises, worksheets
Gershkovich [42]	Not specified	Yes	8	8	Yes	Multimedia, interactive exercises, homework, quizzes
Gershkovich [43]	Not specified	Yes	8	8	Yes	Multimedia, interactive exercises, homework, quizzes
Hesser [44]	Not specified	Yes	8	8	Yes	Multimedia, interactive exercises, homework
Hoffmann [45]	Not specified	Yes	7	12	Yes	Multimedia, interactive exercises, homework
Kelson [46]	FearLess	No	9	2	No	Text, images, interactive exercises, quizzes
Levin [47]	ACT on College Life	No	2	3	No	Multimedia, animations, interactive exercises, worksheets, quizzes
Levin [48]	ACT on College Life	No	3	4	Yes	Multimedia, animations, interactive exercises, worksheets, quizzes
Levin [49]	ACT on College Life	No	2	3	No	Multimedia, animations, interactive exercises, worksheets, quizzes
Levin [50]	Not specified	No	6	4	No	Multimedia, interactive exercises, homework, worksheets,
Ljótsson [51]	Not specified	Yes	5	10	Yes	Text, interactive exercises, worksheets
Molander [52]	Not specified	No	8	8	Yes	Text, images, audio, interactive exercises, homework
Pots [53]	Not specified	Yes	9	12	Yes	Multimedia, interactive exercises, diary
Räsänen [54]	The Student Compass	No	9	7	Yes	Multimedia, interactive exercises, journal
Trompetter [55]	Living with Pain	Yes	9	12	Yes	Text, interactive exercises, diary
Viskovich [56]	You Only Live Once	No	4	4	No	Multimedia, interactive exercises, animations

^a^Multimedia: combined use of audio, video, text, and images.

**Table 4:** Details of attrition rates and treatment satisfaction.

Source	Attrition (%)	ITT^a^	Satisfaction measures	Satisfaction outcomes
Buhrman [37]	17.14	Yes	—^b^	—
Carlbring [38]	0	Yes	—	—
Dahlin [39]	19.2	Yes	NSQ^c^	High satisfaction
Dahlin [40]	0	Yes	CSQ-8^d^	High satisfaction
Fiorillo [41]	16	Yes	CSQ-8	High satisfaction
Fiorillo [41]	16	Yes	SUS^e^	High satisfaction
Gershkovich [42]	0	Yes	NSQ	High satisfaction
Gershkovich [43]	31	Yes	NSQ	High satisfaction
Hesser [44]	8.57	Yes	—	—
Hoffmann [45]	20	No	—	—
Kelson [46]	26	No	SUS	Above average satisfaction
Levin [47]	8	Yes	SUS	High satisfaction
Levin [48]	56	Yes	SUS	High satisfaction
Levin [48]	56	Yes	NSQ	High satisfaction
Levin [49]	30	Yes	SUS	Above average satisfaction
Levin [50]	20	Yes	SUS	Above average
Levin [50]	20	Yes	NSQ	Adequate satisfaction
Ljtósson [51]	2	Yes	—	—
Molander [52]	16.13	Yes	—	—
Pots [53]	15	Yes	—	—
Räsänen [54]	12	Yes	NSQ	High satisfaction
Trompetter [55]	28.05	Yes	CSQ-8	High satisfaction
Trompetter [55]	28.05	Yes	NSQ	More than adequate satisfaction
Viskovich [56]	60.77	Yes	SUS	Above average satisfaction

^a^ITT: intention-to-treat analysis.

^b^not applicable.

^c^NSQ: nonstandardized questions.

^d^CSQ-8: Client Satisfaction Questionnaire-8 items.

^e^SUS: System Usability Scale.

**Table 5:** Details on study quality, anxiety measures, outcomes, and effect sizes (Cohen *d*) for internet-delivered Acceptance and Commitment Therapy (iACT).

Source	POMRF^a^	Measures	WG^b^ effect size	BG^c^ effect size
Buhrman [37]	23	HADS-A^d^	—^e^	0.18 (iACT vs WLC^f^)
Carlbring [38]	22	BAI^g^	—	0.45 (iACT vs WLC)
Dahlin [39]	23	PSWQ^h^	1.35 (pre^i^ to post^j^)	0.87 (iACT vs WLC)
Dahlin [39]	23	GAD-7^k^	1.89 (pre to post)	0.98 (iACT vs WLC)
Dahlin [39]	23	GAD-Q-IV^l^	1.5 (pre to post)	0.70 (iACT vs WLC)
Dahlin [39]	23	BAI	0.92 (pre to post)	0.55 (iACT vs WLC)
Dahlin [39]	20	PSWQ	2.14 (pre to post)	—
Fiorillo [41]	20	DASS-A^m^	0.89 (pre to post)	—
Gershkovich [42]	18	SPAI-SP^n^	1.47 (pre to post)	—
Gershkovich [42]	18	LSAS^o^	0.92 (pre to post)	—
Gershkovich [43]	22	SPAI-SP	1.07 (pre to post)	NS^p^
Gershkovich [43]	22	LSAS	0.77 (pre to post)	NS
Gershkovich [44]	22	SIAS^q^	1.01 (pre to post)	NS
Hesser [44]	29	HADS-A	—	0.59 (iACT vs Forum)
Hoffmann [45]	22	WI-7^r^	1.63 (pre to post)^s^	—
Hoffmann [45]	22	SCL-92 Anx^t^	0.73 (pre to post)^s^	—
Kelson [46]	11	DASS-A	0.42 (pre to FU^u^)	—
Kelson [46]	11	GAD-7	0.66 (pre to post)	—
Levin [47]	13	DASS-A	0.95 (pre to FU)	0.81 (iACT vs WLC)
Levin [48]	14	DASS-A	0.55 (pre to post)	—
Levin [49]	11	DASS-A	NS	NS
Levin [50]	13	CCAPS-Anx^v^	0.39 (pre to post)	NS
Levin [50]	13	CCAPS-Social	0.69 (pre to post)	0.78 (iACT vs WLC)
Ljótsson [51]	18	HADS-A	0.75 (pre to post)	—
Molander [52]	17	GAD-7	—	NS
Pots [53]	27	HADS-A	—	0.49 (iACT vs WLC)
Pots [53]	27	HADS-A	—	0.41 (iACT vs EW^w^)
Räsänen [54]	19	DASS-A	0.42 (pre to post)	*Ns* (iACT vs WLC)
Trompetter [55]	28	HADS-A	—	0.39 (iACT vs WLC)
Trompetter [55]	28	HADS-A	—	*Ns* (iACT vs EW)
Viskovich [56]	10	DASS-A	0.32 (pre to post)	—

^a^POMRF: psychotherapy outcome study methodology rating form.

^b^WG: within-group.

^c^BG: between-group.

^d^HADS-A: Hospital Anxiety and Depression Scale-Anxiety subscale.

^e^N/A: not applicable.

^f^WLC: wait-list control.

^g^BAI: Beck Anxiety Inventory.

^h^PSWQ: Penn State Worry Questionnaire.

^i^pre: pretest.

^j^post: posttest.

^k^GAD-7: Generalized Anxiety Disorder-7 item.

^l^GAD-Q-IV Generalized Anxiety Disorder Questionnaire IV.

^m^DASS-A: Depression, Anxiety and Stress Scales-Anxiety subscale.

^n^SPAI-SP: Social Phobia and Anxiety Inventory-Social Phobia subscale.

^o^LSAS: Liebowitz Social Anxiety Scale-Total.

^p^NS: nonsignificant.

^q^SIAS: Social Interaction Anxiety Scale.

^r^WI-7: Whiteley Index-7.

^s^Standardized response mean effect size.

^t^SCL-92: Symptom Checklist-92.

^u^FU: follow-up.

^v^CCAPS: Counseling Center Assessment of Psychological Symptoms.

^w^EW: expressive writing.

## Discussion

### Overview

In total, 18 of the 20 studies found in this systematic review reported small-to-large anxiety reductions among participants after iACT on standardized measures. Studies typically aimed to treat a primary issue other than an anxiety disorder. Just over half were RCTs, with a fewer number of randomized or uncontrolled pilot studies of new iACT interventions. The interventions were typically rich in multimedia information content and interactive exercises that were provided to participants with therapist guidance over a period of several weeks. Attrition rates were commensurate to other website interventions based on other therapy models [32], and treatment satisfaction was rated above average to high among participants who completed their respective iACT program. There is still considerable need to test iACT on all anxiety conditions with studies of greater methodological quality to corroborate extant findings.

### Participant Characteristics

The delivery of iACT for anxiety is a new field as all studies identified in this review were published from 2012 onward. Many studies were pioneering new iACT programs for people in their respective countries, with Sweden and the United States featured prominently. There were few to no identified studies from other major Western countries such as the United Kingdom, Australia, New Zealand, and Canada. Participants in the included iACT studies were primarily young to middle-aged adults with a high representation of females, indicating that there is substantial need to improve the generalizability of results by creating or applying existing iACT interventions to the wider population across other demographics (eg, children, adolescents, the elderly, and male participants).

### Research Designs

Nine of the iACT studies were uncontrolled pilot studies. Preliminary intervention testing with open and uncontrolled research designs has merit for cost-effectively recruiting participants and demonstrating feasibility of the program on key issues of usability, safety, and limited efficacy [34]. Future creators of iACT programs can also glean insight from these studies on how to design, develop, and test their new interventions for phase I clinical trials. However, uncontrolled pilot studies do not allow for solid conclusions on the efficacy of treating anxiety with iACT because of the lack of control for confounding factors, such as concomitant treatments. For instance, the effect sizes of the uncontrolled studies may be inflated as they are susceptible to threats of internal validity such as maturation, history, and regression to the mean [17]. Therefore, the magnitude of the reported effect sizes in this review cannot be compared across uncontrolled and controlled studies. The remaining studies had stronger research designs that used wait-list control and active treatment groups for comparators. Overall, more RCTs are required to corroborate the promising findings of the uncontrolled pilot studies.

### Internet-Delivered Acceptance and Commitment Therapy Interventions

Presently, there is no single or leading iACT intervention for anxiety. Multiple programs have been developed and tested with key differences. For instance, some iACT programs were tailored with information and exercises to people from specific groups such as university students [47], or chronic pain sufferers [55]. Few iACT interventions had more than one study investigating their therapeutic impact. Due to the heterogeneity between programs, it is not clear what modules were well received by participants and facilitated therapeutic change. Future corelational and component analytical studies on which modules/exercises are better suited for certain populations could help identify optimal features and predictors of success between interventions [13].

Despite the differences between iACT interventions, there were still commonalities. This included the multimedia representation of content, proposed mechanisms of change underlying exercises (eg, acceptance and mindfulness), number of intervention modules, and overall treatment length of several weeks. Another key shared factor was therapist guidance, with most studies having used therapists to varying extents. Research has reported that therapist-guided interventions, in general, produce larger effects than unguided interventions [20]. However, Gershkovich et al [43] found equal anxiety reductions delivering iACT with or without therapist support. Furthermore, RCT studies by Levin et al [47,50] found significant reductions in subclinical anxiety symptoms with unguided iACT. These outcomes should be examined in further research. If the findings hold across studies with greater methodological rigor, then unguided iACT could potentially help address therapist worker shortages [22,23].

### Attrition and Treatment Satisfaction

Attrition rates in the active treatment phase of iACT varied considerably between studies. Three studies in particular had a high percentage of participants not completing the entire program [38,48,56]. Carlbring et al [38] used no treatment satisfaction measures for their iACT program, so it was not possible to gauge the extent to which this impacted upon adherence. In contrast, Levin et al [48] had high system usability and treatment satisfaction ratings from program completers despite a high attrition rate. Similar positive ratings were also received across all other studies that included treatment satisfaction, measures regardless of attrition rate. This suggests that iACT may not be suitable for everyone but can be particularly well received by some as an online treatment modality. The mean attrition rate across all included studies (19.19%) was lower than the median dropout rate of 24% identified across other Web-based interventions, and lower than the weighted average of 35% [32]. Future research would need to make equivalency comparisons on attrition rates between iACT programs and mainstream iCBT programs to help determine if iACT has superior user retention.

### Anxiety Outcomes

#### Generalized Anxiety Disorder

Two studies indicated that iACT can be efficacious and acceptable for adults with GAD [39,40]. The first study by Dahlin et al [40] found a large reduction on GAD symptomatology across 14 participants. However, this uncontrolled pilot study had a small sample, which limits conclusions on the true efficacy of *The Worry Help* program because of possible treatment confounds. However, large reductions across multiple measures of GAD were also found in a subsequent RCT study by Dahlin et al [39]. There was no clinically significant deterioration throughout this study, with GAD symptoms improving beyond the acute treatment phase [40]. A key limitation of this study, however, was the use of only a wait-list control rather than an active treatment group. Nevertheless, both of these studies still provide early support for treating adults with GAD using therapist-guided iACT over a period ranging from 8 to 10 weeks. This pattern of results mirrors the large GAD anxiety reductions of face-to-face ACT treatment [12], which implicates the potential of guided iACT to provide adjunctive clinical care.

#### Social Anxiety Disorder

Two studies indicate that iACT can be efficacious and acceptable for adults with SAD [42,43]. The first study by Gershkovich et al [42] found large effects on multiple measures of SAD symptoms across 13 participants. However, this uncontrolled pilot study had a small sample, which limits conclusions on the true efficacy of this iACT program because of possible treatment confounds. The other study by Gershkovich et al [43] investigated the same iACT program with participants randomized to 1 of 2 conditions: treatment with or without therapist support. Once more there were large reductions in SAD anxiety symptoms found in both groups at posttest with 14 out of 26 (53.8%) participants no longer meeting SAD criteria. Key limitations of this study were the lack of a non-iACT control group, and no participant follow-up assessments. These foundational studies provide tentative support for treating adults with SAD using iACT over an 8-week period. This pattern of results mirrors the large SAD anxiety reductions found with face-to-face ACT treatment [12], which further implicates the potential of iACT to provide adjunctive clinical care.

#### Illness Anxiety Disorder

It appears that iACT holds promise as an efficacious treatment for IAD, a condition of severe health anxiety that was previously known as hypochondriasis before the DSM-5 [45]. Significantly large reductions on both measures of anxiety were found at post-treatment that were also sustained at 3-month follow-up. This implies that therapist-guided iACT over 12 weeks is a feasible treatment approach for severe health anxiety among adults. However, this study was an uncontrolled pilot study with a small sample size of 15 self-referred patients. Therefore, these preliminary results must be validated in further larger-scale RCT research to control for treatment confounds.

#### General Anxiety Symptoms

Small-to-large improvements in general anxiety symptoms were reported across most studies that evaluated iACT on various physical and mental health issues. These outcomes were found across several different Western countries, which indicate the capacity for iACT to transcend national and cultural borders. Furthermore, the highest quality RCT evidence in this review revealed that iACT was just as efficacious as iCBT [44], and expressive writing [53,55]. These studies had the highest POMRF scores in the review with a range of 27 to 29. These scores are all well above the average POMRF score of 18.1 reported earlier for face-to-face ACT treatment RCTs [36]. Additionally, iACT outcomes were maintained at follow-up for all studies that found significant reductions. The primary implication is that iACT can provide a transdiagnostic, efficacious, and acceptable treatment option for people with general anxiety symptoms.

However, the results of studies that investigated the benefit of iACT for general anxiety symptoms, specifically among young adult university students, were mixed. Several studies indicated that iACT can be efficacious for reducing anxiety symptoms in this population with moderate-to-large effects [46-48,50]. Results in 2 of these studies [47,48] were procured from online treatment using fewer ACT-based modules (2 to 3) than the other iACT studies (5 to 9). This suggests that it could be possible to streamline iACT content to produce equivalent results to programs with more content. Furthermore, 2 of these studies [46,47] had no therapist guidance, and the pilot study by Kelson et al [46] found significant subclinical anxiety reduction after 2 weeks. These findings hold promise for delivering unguided iACT interventions in the form of self-help, or stepped care, to the “worried well” to procure rapid anxiety relief [6].

However, while the RCT by Räsänen et al [54] found a moderate within-group effect of guided iACT treatment on anxiety reduction in young adults, there was no significant difference between the iACT condition and the wait-list control group at posttest. Furthermore, an RCT by Levin et al [49] did not find a significant anxiety reduction after delivering 2 modules of unguided iACT treatment over a 3-week period. This outcome contradicts the significant results of the earlier RCT study by Levin et al [47] that delivered the same iACT intervention without therapist guidance over 3 weeks. Together, these results imply that iACT may not be efficacious for all young adults, or that possible confounds occurred, such as wait-list participants using concomitant treatments, or natural fluctuations in subclinical symptomatology.

It is important to note that all of the unguided iACT studies that included participants with subclinical anxiety had low POMRF ratings. Therefore, methodological issues limit conclusive interpretations of these early stage outcomes. For instance, the first study by Levin et al [47] was a small feasibility trial that used a less conservative alpha level to determine significance (*P*<.10). The studies by Kelson et al [46] and Levin et al [48] made use of single-group, uncontrolled open-trial designs. The third study by Levin et al [49] had low power and did not use a dosage equivalent treatment condition to help control for nonspecific confounds. Pilot study recruitment issues of university students may also have affected results. For instance, most participants in the most recent study by Levin et al [50] reportedly sought the incentive of psychology course credits more so than mental health treatment which could have impacted upon observed program engagement, satisfaction, and self-report outcomes. Therefore, subsequent studies would need to appraise the impact of iACT in university settings among a pure treatment seeking a sample that does not receive any inducements such as money or course credits. Despite these limitations, there is still a need for more iACT research on people suffering from subclinical general anxiety symptoms, as they can be quite debilitating [5]. Therefore, more methodologically rigorous RCT studies are needed to clarify the utility of unguided iACT for subclinical anxiety.

### Future Research

Presently, there is substantial need to test iACT interventions on all anxiety-related problems. There were no iACT studies on separation anxiety disorder, selective mutism, specific phobia, panic disorder, agoraphobia, substance/medication-induced anxiety disorder, or anxiety disorders due to medical conditions [2]. Questions also remain regarding the capacity of iACT interventions to enact therapeutic change in vein of established treatments, such as iCBT, on multiple factors (eg, cost-effectiveness, credibility, brevity, outcome longevity, and integration with clinical services). Therefore, further investigation into the impact of individual ACT-based modules, with or without therapist guidance, using RCT designs that incorporate an active positive control is warranted. This could help pinpoint optimal treatment time lengths, clarify the quality of content, and elucidate the utility of unguided treatment protocols. Future research should also consider quality appraisal guidelines such as Öst’s [36] POMRF criteria when designing iACT studies. This would help address gaps in the current literature, such as concomitant treatment confounds, lack of clinical significance reporting, and anxiety outcome sustainability beyond a year. As most of the iACT programs were researched by the people who developed them, independent investigation would clarify the validity of extant findings.

### Review Limitations

This systematic review has several limitations. Owing to the inclusion of only peer-reviewed English-language journal articles, there may potentially be more anxiety iACT studies in the public domain available in other languages or formats (eg, conference proceedings). Even among the English language–based literature reviewed, there were studies found which met inclusion criteria, yet were not self-described as iACT. For example, several studies used terms such as “acceptance-based” or “acceptance and values-based behavior therapy” in their article titles [38-40,51]. It is possible that a study may have been overlooked because of obscure nomenclature. Also, this systematic review only focused on Web-based interventions and did not include in its scope other online modalities, such as mobile phone apps, social media, and virtual reality systems. Furthermore, there were no subgroup analyses performed on the impact of iACT on anxiety. Thus, insight was lacking into the applicability of iACT to people with specific demographic characteristics (eg, gender, age, or education level).

With regard to quality appraisal limitations, all types of studies were included in this review to reduce publication bias. Although non-RCT studies can provide insight into iACT on anxiety, there is a greater risk of bias that needs to be accounted for when interpreting the validity of the reported effects. This risk of bias was addressed with Öst’s [36] POMRF questionnaire in this review, yet there is still room for subjectively interpreting POMRF criteria. For example, a study may have actually met certain criteria in its execution, yet scored lower on an item in our quality appraisal because of the authors omitting that information in the publication text. Although the subjective interpretation of risk of bias is an issue with any quality appraisal method, it is still a limitation to consider that may impact on the capacity to replicate the findings of this systematic review.

### Conclusions

Nearly all studies reported the beneficial impact of iACT on anxiety. Small to large reductions in anxiety symptoms among populations suffering from GAD, SAD, IAD, and anxiety-related health problems were found in all but 2 studies. More research is required to establish outcomes on iACT for other anxiety relevant conditions such as panic disorder, agoraphobia, separation anxiety, selective mutism, and specific phobias [2]. An important aspect of this is making direct comparisons of iACT treatments with established active interventions (eg, iCBT) on key standardized measures of anxiety, adherence, usability, and treatment satisfaction. Further research on iACT for anxiety on untested demographics (eg, children, adolescents, and the elderly), as well as in countries and cultural settings outside of Sweden and the United States, is also warranted. Overall, the current findings indicate that iACT can be an effective and acceptable treatment for some anxiety conditions among young to middle aged adults in Western societies.

## Appendix

Multimedia Appendix 1 Preferred Reporting Items for Systematic Reviews and Meta-Analyses checklist.

## Abbreviations

ACBS: Association for Contextual Behavioral Science
ACT: Acceptance and Commitment Therapy
CBT: cognitive behavioral therapy
CSQ-8: Client Satisfaction Questionnaire-8 Items
DSM-5: Diagnostic and Statistical Manual of Mental Disorders-Fifth Edition
GAD: generalized anxiety disorder
iACT: internet-delivered Acceptance and Commitment Therapy
IAD: illness anxiety disorder
iCBT: internet-delivered Cognitive-Behavioral Therapy
NSQ: nonstandardized questions
POMRF: psychotherapy outcome study methodology rating form
PRISMA: Preferred Reporting Items for Systematic Reviews and Meta-Analyses
RCT: randomized controlled trial
SAD: social anxiety disorder
SUS: System Usability Scale
